# CANNUSE, a database of traditional Cannabis uses—an opportunity for new research

**DOI:** 10.1093/database/baab024

**Published:** 2021-05-01

**Authors:** Manica Balant, Airy Gras, Francisco Gálvez, Teresa Garnatje, Joan Vallès, Daniel Vitales

**Affiliations:** Institut Botànic de Barcelona (IBB, CSIC-Ajuntament de Barcelona), Passeig del Migdia s/n, Barcelona, Catalonia 08038, Spain; Institut Botànic de Barcelona (IBB, CSIC-Ajuntament de Barcelona), Passeig del Migdia s/n, Barcelona, Catalonia 08038, Spain; Centro de Investigación y Desarrollo de Recursos Científicos BioScripts, 31, Av. de la Reina Mercedes, Sevilla, Andalusia 41012, Spain; Institut Botànic de Barcelona (IBB, CSIC-Ajuntament de Barcelona), Passeig del Migdia s/n, Barcelona, Catalonia 08038, Spain; Laboratori de Botànica (UB), Unitat Associada al CSIC, Facultat de Farmàcia i Ciències de l’Alimentació-Institut de Recerca de la Biodiversitat (IRBio), Universitat de Barcelona, Avinguda de Joan XXIII, 27-31, Barcelona, Catalonia 08028, Spain; Institut d’Estudis Catalans (IEC), Carrer del Carme, 47, Barcelona, Catalonia 08001, Spain; Institut Botànic de Barcelona (IBB, CSIC-Ajuntament de Barcelona), Passeig del Migdia s/n, Barcelona, Catalonia 08038, Spain; Laboratori de Botànica (UB), Unitat Associada al CSIC, Facultat de Farmàcia i Ciències de l’Alimentació-Institut de Recerca de la Biodiversitat (IRBio), Universitat de Barcelona, Avinguda de Joan XXIII, 27-31, Barcelona, Catalonia 08028, Spain

## Abstract

*Cannabis* is one of the most versatile genera in terms of plant uses and has been exploited by humans for millennia due to its medicinal properties, strong fibres, nutritious seeds and psychoactive resin. Nowadays, *Cannabi*s is the centre of many scientific studies, which mainly focus on its chemical composition and medicinal properties. Unfortunately, while new applications of this plant are continuously being developed, some of its traditional uses are becoming rare and even disappearing altogether. Information on traditional uses of *Cannabis* is vast, but it is scattered across many publication sources in different formats, so synthesis and standardization of these data are increasingly important. The CANNUSE database provides an organized information source for scientists and general public interested in different aspects of *Cannabis* use. It contains over 2300 entries from 649 publications related to medicinal, alimentary, fibre and other uses from different geographical areas and cultures around the world. We believe this database will serve as a starting point for new research and development strategies based on the traditional knowledge.

**Database URL:** http://cannusedb.csic.es

## Introduction

Medicinal plants have almost limitless applications and have traditionally been used to treat several illnesses (1). One of the most commonly used plants is *Cannabi*s, being known to humans for thousands of years and showing myriad traditional uses globally. The oldest known record of its medicinal use dates back to 4700 B.P. in China. Many other ancient texts from India, Persia, Egypt, Greece and Rome also contain valuable information about a plethora of other *Cannabis* medicinal uses (2). In the beginning of the 20th century, *Cannabis* became widely regarded as an illegal drug with negative effects, resulting in a general reduction in its use as a medicine (3). As a consequence—despite the long-standing recognition of its positive effects—the scientific interest in this plant steeply declined for several decades, and most of the information on *Cannabis* use was limited to the domain of local popular knowledge. However, in the last 20 years, interest in *Cannabis* research has grown, and several medicinal uses originally discovered by traditional knowledge have been tested and developed for commercial medicine production (4), modern fibre applications (5) and food production (6–8). The latest boost to scientific and technological interest in *Cannabis* has been the recent decriminalization or legalization of its medicinal and recreational use in many countries, which has boomed into a billion-dollar industry in just a few years (9).

Databases are one of the tools that enable gathering information in an organized repository, which facilitates further research. Several *Cannabis* databases have been created in recent years to collect and organize information related to its genomic resources (10–12), clinical applications (13) or commercial strains (14). However, despite copious information available for this plant, no database on the traditional uses of *Cannabis* has been established so far. The studies on new and traditional uses of *Cannabis* are numerous and are increasing daily; research papers on this topic are being published in many journals from various scientific fields and circulations (i.e., both local and international publications). Therefore, much important information about its uses can stay unnoticed by the majority of people interested in the topic. Another problem that makes the comparison of results difficult is the terminology used. While methods for ethnobotanical studies are well developed, it is up to the authors to decide whether to state the effect of the plant, the target ailment or merely the body system being treated. The lack of data integration and standardization makes it difficult to use this information in research, so synthesis and standardization of all these data are becoming more and more important.

Inventorying traditional knowledge on biodiversity is a way of ensuring its conservation—especially urgent in many zones, where it is being eroded—and its possible further uses for human well-being (15–18). Public databases are a powerful instrument of such traditional knowledge preservation and represent an excellent tool to accomplish one of the ethical exigencies of ethnobotanical prospection: to return the knowledge to the society, where it came from (18). Nevertheless, some concerns have been formulated regarding the role of the current forms of conservation of traditional knowledge, one of them being the scarce implication of its holders in the processes and their difficulties to access the information (17, 19). In this respect, it is important that databases provide open access to, among other users, local communities (15). Here, we present the CANNUSE database (available at http://cannusedb.csic.es), which we have elaborated in order to provide a thorough information source that will be useful for the whole society, including the scientific community, legislators and non-professionals interested in any aspect of *Cannabis* use. We have undertaken a comprehensive data collection that enabled us to construct the first global database on *Cannabis* medicinal, alimentary, fibre, psychoactive and other ethnobotanical uses. The main aim of the CANNUSE database is to gather and organize this abundant information on traditional *Cannabis* use in a simple manner. Therefore, we believe that the user-friendly web interface constructed for the database will enable easy access to this reserve of information to any type of public and return the knowledge back to society. We hope this resource will facilitate research and development strategies for drug, food or other *Cannabis* products based on traditional knowledge and enable legislators to take decisions on relevant legal dispositions. The database will also serve to bring to light lesser-known traditional *Cannabis* uses (medicinal and others) that have been thus far overlooked and may be disappearing, enhancing new ethnobotanical studies and perhaps promoting beneficial applications of some of these rare uses. In addition, this organized repository of *Cannabis* data may help detect less obvious connections between specific plant parts and illnesses, which could open novel treatment lines based on *Cannabis* products.

## Methods

### Publication search

**Figure 1.**
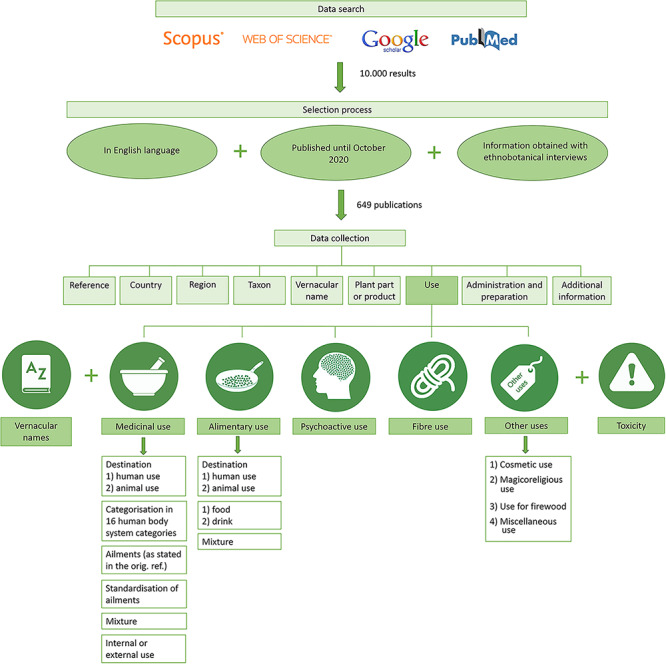
Workflow of the data search, selection process and data collection for the CANNUSE database construction.

Our publication search was carried out in four major online databases—Scopus, Web of Science, PubMed and Google Scholar, using the following set of keywords and exact terms: *Cannabis* AND (‘folk medicine’ OR ‘traditional medicine’ OR ‘ethnobotany’ OR ‘traditional knowledge’). Our search returned over 10000 results (Figure 1).

During the screening process, we eliminated references that (i) were not published in English language, (ii) were not published by the end of October 2020 and (iii) did not obtain the information included through ethnobotanical interviews. To avoid duplication, information obtained from review papers and books was only used when original research papers could not be found. In further steps, papers containing inconsistencies (e.g. incorrect citations, unclear uses and uses in review papers not matching with the original research papers) were also eliminated. Additional references cited in relevant ethnobotanical papers were added using the snowball method (20).

After filtering and excluding the papers that did not fit our criteria, we obtained a final reference list with 649 publications. Most of these were research papers (607 references, including 6 conference proceedings), but 38 review papers, 2 doctoral theses and 2 master theses were also included. We registered a total of 2330 data entries on traditional uses of *Cannabis*. One data entry is represented by one use quoted in a publication.

### Data collection

For each reference, the following information was recorded (Figure 1): (i) type and year of publication, (ii) country, (iii) region, (iv) taxon, (v) vernacular names and (vi) part of the plant (inflorescence, leaf, whole plant, seed, aerial part, stem, bark, root, twig and branch, and other part) or plant product (resin, fibre and other product) used. In this database, the term ‘seed’ actually refers to the *Cannabis* fruit—a nut (also called achene) (21). In the reviewed literature, this part was referred to with several terms (fruit, young fruit, nut, achene and seed); because the term ‘seed’ was the most commonly used and generally accepted, the part was referred to in the database under the single term. We also recorded (vii) use categories (medicinal, alimentary, fibre, psychoactive or other), whether *Cannabi*s had (viii) animal or human use, if the plant was considered (ix) toxic or noxious (toxicity) or included (x) modes of preparation and administration, whenever they were provided by the authors. When other ingredients (plant, animal or other substance) were added to the alimentary and medicinal preparations, they were categorized as a (xi) mixture. For medicinal uses, (xii) the way of administration (external and internal) was also recorded when possible. Any additional information available (xiii) was also recorded. When vernacular names related to the use of the plant were provided (disease names, names of the recipes or products, etc.), these were included within square brackets.

### Structure of the CANNUSE database

The database is structured in five use categories: medicinal (which includes also veterinary), alimentary, fibre, psychoactive and other use. We also added a category for toxicity reports and one for vernacular names (Figure 1). Each database entry was attributed to one or more appropriate categories. For instance, in the case of a reference stating ‘traditional drink “thandai” which has a sedative effect and is narcotic’, the entry was included in three categories: medicinal use (sedative), alimentary use [drink (*thandai*)] and psychoactive use (narcotic).

Detailed information on CANNUSE categories is described below:

#### Medicinal use

Medicinal use was divided into human and animal medicinal use. Depending on the reference, the uses were originally formulated in many different ways—sometimes via a name of the relevant disease or condition treated with *Cannabis* (e.g. diabetes) and other times by its putative effect (e.g. antidiabetic). To simplify the search and access to the data, we standardized all the use reports, so they refer the plant’s effect, and renamed them according to the Oxford Concise Medical Dictionary (22), but the original use (as stated in the paper) was also retained for easy verification. To make searching faster, human medicinal uses were classified into 16 human body system categories, according to Cook (23) with minor modifications. Categories used in the database are circulatory system and blood disorders, digestive system and nutritional disorders, endocrine system and metabolic disorders, genitourinary system disorders, immune system disorders and neoplasia, infections and infestations, musculoskeletal system disorders and traumas, nervous system and mental disorders, pain and inflammations, poisoning, pregnancy, birth and puerperal disorders, respiratory system disorders, sensory system disorders, skin and subcutaneous tissue disorders, tonic and restorative, and unclassified. Sometimes one use could belong to two system categories (e.g. bladder inflammation is placed under two system categories—pain and inflammations and genitourinary system disorders). Veterinary uses were not further divided into system categories.

#### Alimentary use

Alimentary use was divided into human and animal use, and again into food and drink categories. Traditional drinks containing *Cannabis*, which had medicinal, psychoactive or religious uses, were automatically added into the alimentary use category even if this use was not additionally specified.

#### Fibre use

This category contains information on *Cannabis* use for the production of fabric, rope, sack and other products and was not divided further. It only contains information on human uses.

#### Psychoactive use

The category psychoactive use includes reports related to ‘narcotic’, ‘intoxicating’ and other effects altering perception, mood or consciousness. The term ‘narcotic’ can be defined as ‘a drug or other substance that affects mood or behaviour and is consumed for non-medical purposes, especially one sold illegally’ or as ‘a medical drug that relieves pain and induces drowsiness, stupor, or insensibility’ (24). A precise interpretation of the term in publications was not always possible, hence all ‘narcotic’ references were classified into the category psychoactive use. The category only contains information on human uses.

#### Other uses

The remaining, less-numerous uses were placed in category other uses, which was further separated into four lower categories: cosmetic, magicoreligious, firewood and miscellaneous use. The category only contains information on human uses.

#### Toxicity

Even though in most regions plants from the genus *Cannabis* are considered valuable medicinal plants, in certain regions of the world they are considered toxic, with their consumption (or prolonged consumption and abuse) causing several side effects (e.g. diarrhoea, nausea, poisoning, etc.). All these reports were assigned to this category.

#### Vernacular names

Wild, cultivated or commercialized, *Cannabis* is widely distributed around the world, and many of its parts are used for a variety of purposes. For these reasons, it has not only been popularly named in many languages, but often with several terms in each one, depending on the part, product or use (cf., just in English, hemp, cannabis and marijuana). For the majority of references, the authors provided vernacular names for *Cannabis* and these are included here.

## Quick overview of the data records

The database contains 2330 data entries on medicinal, alimentary, fibre, psychoactive and other ethnobotanical uses of *Cannabis* from different geographical areas and cultures worldwide. It contains information on the intended purpose of plant use, the taxonomic and vernacular name, the country and region of use, bibliographic reference type, plant part used, intended use destination (human or animal use), details on preparation and administration and any other additional information we considered important. Each entry is connected to the original source, which can be accessed easily from the website.

**Figure 2.**
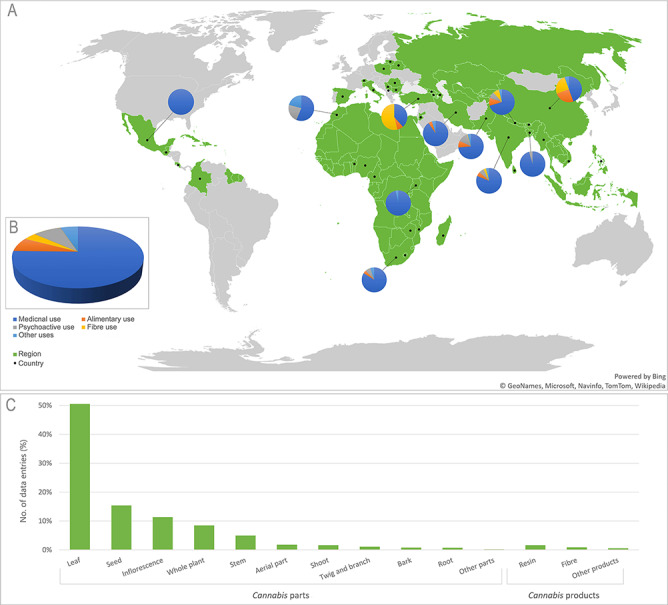
(A) Map of world regions (green) and countries (•) represented in the CANNUSE database, with pie charts showing distribution of uses in the countries with over 50 records. The background map was produced using the Excel Office. (B) Distribution of *Cannabis* uses presented in the database. (C) Distribution of *Cannabis* parts and products presented in the CANNUSE database (in %).

**Figure 3.**
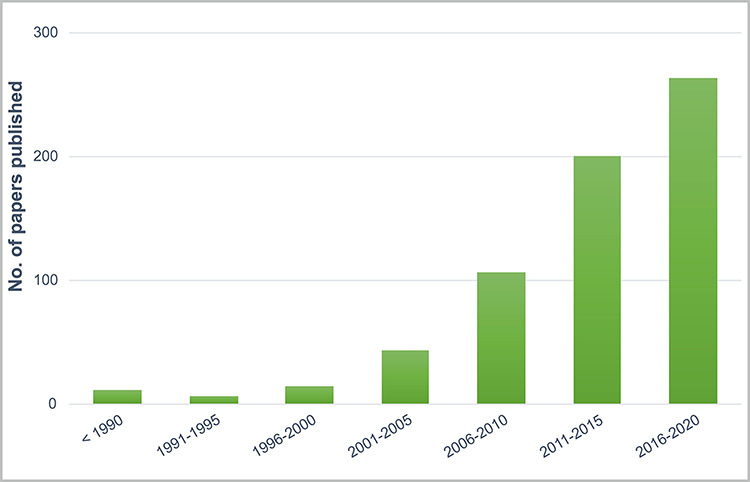
Number of publications in CANNUSE database containing information about *Cannabis* ethnobotanical uses published over the years.

Information was gathered from 649 references from 41 countries worldwide (Figure 2A). The majority of them (71.98%) were published in the last 10 years (Figure 3). Reports from India (41.76%) and Pakistan (25.89%), where the use of *Cannabis* in folk medicine has a long cultural tradition (25–27), represent the greatest proportion of entries in the database. Most of the reported uses were medicinal (75.41%), followed by psychoactive (8.35%), alimentary (7.29%), other uses (5.13%) and fibre use (3.82%) (Figure 2B). The most frequently used plant parts are leaf (50.51%), seed (15.38%) and inflorescence (11.35%), while other plant parts represent a smaller proportion (Figure 2C). We identified *Cannabis* treatments for 210 human and 53 animal ailments. Reports on its toxicity only represent 3.24% of data entries.

## User guide and potential data applications

The CANNUSE database is openly accessible at http://cannusedb.csic.es. Besides the web interface, we also provide the data via the DIGITAL.CSIC repository (https://digital.csic.es/handle/10261/226973?mode=full; Table 1), where CANNUSE database can be downloaded as a Microsoft Excel file, under the terms of a Creative Commons Attribution-NonCommercial-ShareAlike 4.0 (CC BY-NC-SA 4.0) International License.

Search through the database is facilitated by a user-friendly graphical interface. The clean design used is visible from any type of device (e.g. smartphones, laptops and tablets), easy to use and without page reloads so the visitor can use the search quickly and efficiently.

**Figure 4.**
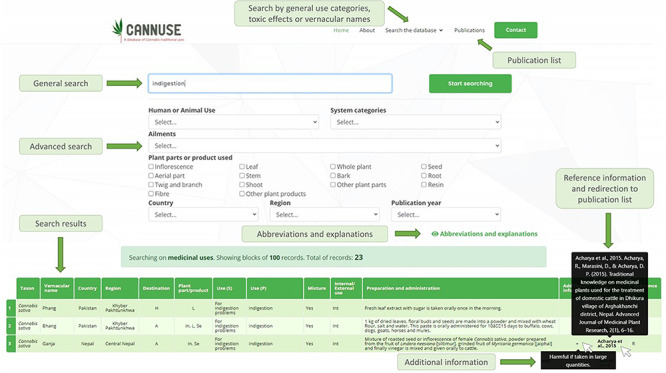
User interface of the Search the database function. The data can be filtered out using general search (search by key words) or by advanced search, where user is selecting the filters for the search.

The web version of the database offers two search options (Figure 4). The first one is a general search, based on key words, while the second is an advanced search where additional filter options are available (plant parts and products, country, region, year of publication, etc.) depending on the general category being selected. Due to the limited space available in the graphical interface, abbreviations are used for more than one field, but their explanations can be quickly located in the ‘Abbreviations and explanations’ section of the website. Where additional information is available, the movement of the cursor over the sign ‘+’ reveals additional text. Original references are connected to each entry and are linked to the tab ‘Publications’, where full reference information and links to the original publications can be found. Movement of the cursor over the short version of the citation reveals the full reference information. Search results are obtained in a table below the menus.

The CANNUSE database offers an organized and structured dataset that can be used as a basis for research and development strategies in many different scientific and technological fields. For example, at the present time, the majority of medical studies are focused on the application of *Cannabis* inflorescences for new treatments, but traditionally, many other parts of the plant were used for the treatment of different conditions and ailments. The CANNUSE database enables us to filter down to specific plant parts and identify the corresponding ailments for which they have been traditionally used. Furthermore, new applications could be developed in food and nutraceutical, cosmetic or recreational use industries. Analysis of *Cannabis* medicinal (and other) uses in different regions of the world could indicate local variability in *Cannabis* landraces, which would make them more suitable for further development into specific medicines. Furthermore, ethnobotanical records in the CANNUSE database could be considered as relevant additional information (besides genetic diversity and archeological findings) that could help determine the origin of species and its dispersion history (28).

The CANNUSE database contributes to the conservation and dissemination of many traditional uses in many parts of the world of an emblematic plant in ethnobotany and economic botany. It protects the traditional knowledge holders from misappropriation of their knowledge, using a CC BY-NC-SA 4.0 International License, which allows for sharing and adaptation of the database, with appropriate crediting, but does not permit its use for commercial purposes. Much of the ethnobotanical reports (databases and academic papers) are published in English, which enables a bigger distribution of the knowledge but diminishes their usability for original traditional knowledge holders (16). The CANNUSE database is, indeed, in English, but now powerful translation tools are easily available for many languages. In addition, we have already started to look for publications in other languages for further updates of the database. In this step, we are planning to include local scientists, which would enable us the contact with traditional knowledge holders missing so far.

## Future development

The CANNUSE database already contains a comprehensive and globally distributed dataset on traditional *Cannabis* uses. However, several potential areas for upgrade have already been identified. The data included in this version were obtained only from references written in English, while publications in other languages were up to this point excluded. Ethnobotanical research is often published in lesser-known, local journals, which are not written in English, so many additional uses remain to be included in future updates planned. To improve the protection of the traditional knowledge holders’ rights and facilitate their benefit-sharing claims, the information about the ethnic group where the use comes from will be added. The database will be updated annually with new literature and subsequently information on *Cannabis* uses from historic sources, books, review papers and other secondary sources, and sources in languages other than English. Additional information gathered with our own ethnobotanical interviews will also be included. Researchers are encouraged to submit any additional data they wish to share through the contact form on the website, to facilitate the improvement of the growing dataset. Regular extensions of the database will ensure that updated information on traditional *Cannabis* uses is thoroughly available for basic and applied research purposes. We ask users to cite this paper when data are used in publications or other activities (e.g. teaching and industrial applications) and to also cite the latest version of the database used.
